# Avian Influenza Virus Infection Risk in Humans with Chronic Diseases

**DOI:** 10.1038/srep08971

**Published:** 2015-03-10

**Authors:** Yaogang Zhong, Yannan Qin, Hanjie Yu, Jingmin Yu, Haoxiang Wu, Lin Chen, Peixin Zhang, Xiurong Wang, Zhansheng Jia, Yonghong Guo, Hua Zhang, Junjie Shan, Yuxia Wang, Hailong Xie, Xiaojie Li, Zheng Li

**Affiliations:** 1 Laboratory for Functional Glycomics, College of Life Sciences, Northwest University, Xi'an, PR China; 2 National Key Laboratory of Veterinary Biotechnology, Harbin Veterinary Research Institute, Chinese Academy of Agricultural Science, Harbin, PR China; 3 Center of infectious diseases, Tangdu Hospital, Fourth Military Medical University, Xi'an, PR China; 4 Department of Oncology, Shaanxi Provincial People's Hospital, Xi'an, PR China; 5 Institute of Pharmacology and Toxicology, Academy of Military Medical Science, Beijing, PR China; 6 Cancer Research Institute, University of South China, Hengyang, PR China

## Abstract

Saliva proteins may protect older people from influenza, however, it is often noted that hospitalizations and deaths after an influenza infection mainly occur in the elderly population living with chronic diseases, such as diabetes and cancer. Our objective was to investigate the expression level of the terminal α2-3- and α2-6-linked sialic acids in human saliva from type 2 diabetes mellitus (T2DM), liver disease and gastric cancer (GC) patients and assess the binding activity of these linked sialic acids against influenza A viruses (IAV). We observed that the expression level of the terminal α2-3-linked sialic acids of elderly individuals with T2DM and liver disease were down-regulated significantly, and the terminal α2-6 linked sialic acids were up-regulated slightly or had no significant alteration. However, in the saliva of patients with GC, neither sialic acid was significantly altered. These findings may reveal that elderly individuals with chronic diseases, such as diabetes and liver disease, might be more susceptible to the avian influenza virus due to the decreased expression of terminal α2-3-linked sialic acids in their saliva.

Influenza A virus (IAV) host specificity is mediated by viral surface hemagglutination (HA), which binds receptors containing glycans with terminal sialic acids (Sia). Avian influenza viruses (AIV) are considered to be key contributors to the emergence of human influenza pandemics, and its HA preferentially recognizes Siaα2-3Galactose (Siaα2-3Gal)-linked receptors^1^,^2^,^3^, whereas human influenza virus HA preferentially recognizes Siaα2-6Gal linked receptors^4^,^5^,^6^. It has been shown that the difference in receptor use between avian and human influenza viruses, combined with the distribution of avian and human influenza virus receptors in the respiratory tract, results in a different localization of virus attachment^7^,^8^. Human influenza viruses attach more abundantly to the upper respiratory tract and trachea, whereas avian influenza viruses predominantly attach to the lower respiratory tract^9^,^10^,^11^.

The oral cavity can be an entry site of the influenza virus, and saliva is known to be a potentially important barrier against IAV infection. The barrier action by saliva can be explained by two distinct mechanisms^12^. One mechanism involves proteins (pulmonary surfactant protein D) binding via carbohydrate-recognition domains to carbohydrates on the HA1 domain of the HA and on the neuraminidase of IAV^13^,^14^. The other mechanism involves proteins (α-2-macroglobulin, MUC5B and salivary glycoprotein-340) inhibiting IAV by presenting a sialic acid ligand for viral HA^15^,^16^,^17^,^18^,^19^. Furthermore, the saliva of healthy elderly individuals is found to contain more Siaα2-3Gal- and Siaα2-6Gal-linked receptors than that of children and adults, which implies that healthy elderly individuals have stronger resistance to IVA partly by presenting more terminal α2-3/6-linked sialic acid residues in their saliva to bind with viral HA and inhibit the activities of IVA^20^. However, it is often noted that hospitalizations and deaths after an influenza infection mainly occur in the elderly population diagnosed with chronic diseases, such as diabetes and cancer^21^,^22^,^23^. This phenomenon is usually explained by the fact that chronic diseases reduce the ability of the immune system to fight infections.

To improve our understanding for the reason why hospitalizations and deaths after an influenza infection mainly occur in the elderly population living with chronic diseases and to find the characteristics of the susceptible population for IVA, we aimed to compare the expression level of the terminal α2-3- and α2-6-linked sialic acids in human saliva from type 2 diabetes mellitus (T2DM), liver disease (hepatitis B (HB), hepatic cirrhosis (HC), hepatocellular carcinoma (HCC)) and gastric cancer (GC) patients and assess the binding activity of these linked sialic acids against IVA.

## Results

### Relative expression levels of terminal α2-3/6-linked sialic acids in human saliva

**Figure 1:**
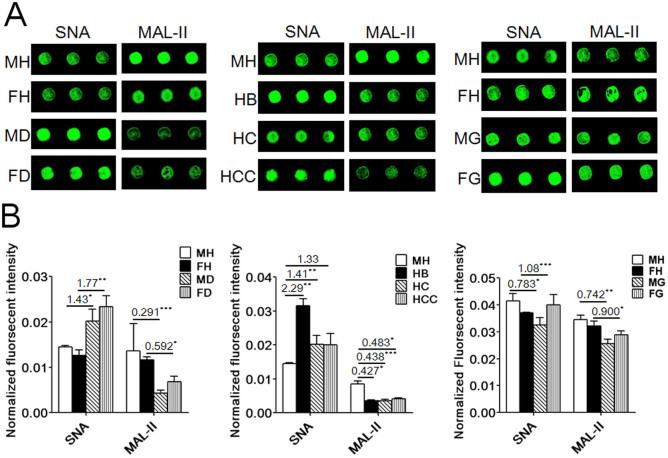
Relative expression levels of the terminal Siaα2-6Gal recognized by SNA and Siaα2-3Gal recognized by MAL-II in human saliva using lectin microarrays. MH: male healthy groups, FH: female healthy groups, MD: male T2DM patients, FD: female T2DM patients, HB: hepatitis B, HC: hepatic cirrhosis, HCC: hepatocellular carcinoma, MG: male GC patients, FG: female GC patients. (A) Fluorescent images from a portion of SNA and MAL-II with three replicates. (B) Normalized fluorescent intensity of SNA and MAL-II from each clinical group compared with healthy groups based on the values of the ratio in pairs (*P < 0.05, **P < 0.01, and ***P ≤ 0.001).

The expression of the terminal Siaα2-6Gal recognized by *Sambucus Nigra Lectin* (SNA) and Siaα2-3Gal recognized by *Maackia Amurensis Lectin II* (MAL-II) were investigated in human saliva from T2DM (Supplementary Table S1), HB, HC, HCC (Supplementary Table S2) and GC patients (Supplementary Table S3) using the lectin microarrays. The images were shown in Figure 1A, and the normalized fluorescent intensities (NFIs) were summarized in Supplementary Table S1, S2 and S3. The NFIs of SNA and MAL-II from each clinical group were compared with healthy groups based on the values of the ratio (fold change >1.50 or <0.67) in pairs and indicated significant (*p*-value less than 0.05) up-regulation or down-regulation of certain glycans. According to the results, the expression levels of the terminal Siaα2-6Gal of female elderly groups with T2DM (fold change >1.5, *p* < 0.01) and the male elderly group with HB (fold change >1.5, *p* < 0.01) were up-regulated significantly. The terminal Siaα2-3Gal of all elderly groups with T2DM (fold change <0.67, *p* < 0.05) and liver disease (fold change <0.67, *p* < 0.05) was down-regulated significantly; however, the terminal Siaα2-6Gal of male elderly groups with T2DM, HC, HCC and GC (0.67< fold change <1.5, *p* < 0.05) had no significant alternation. The expression level of the terminal Siaα2-3Gal of elderly groups with GC (0.67< fold change <1.5, *p* < 0.05) was not significantly altered in saliva (Figure 1B).

### Individual validation of expression levels for α2-3/6-linked sialic acids in saliva

**Figure 2:**
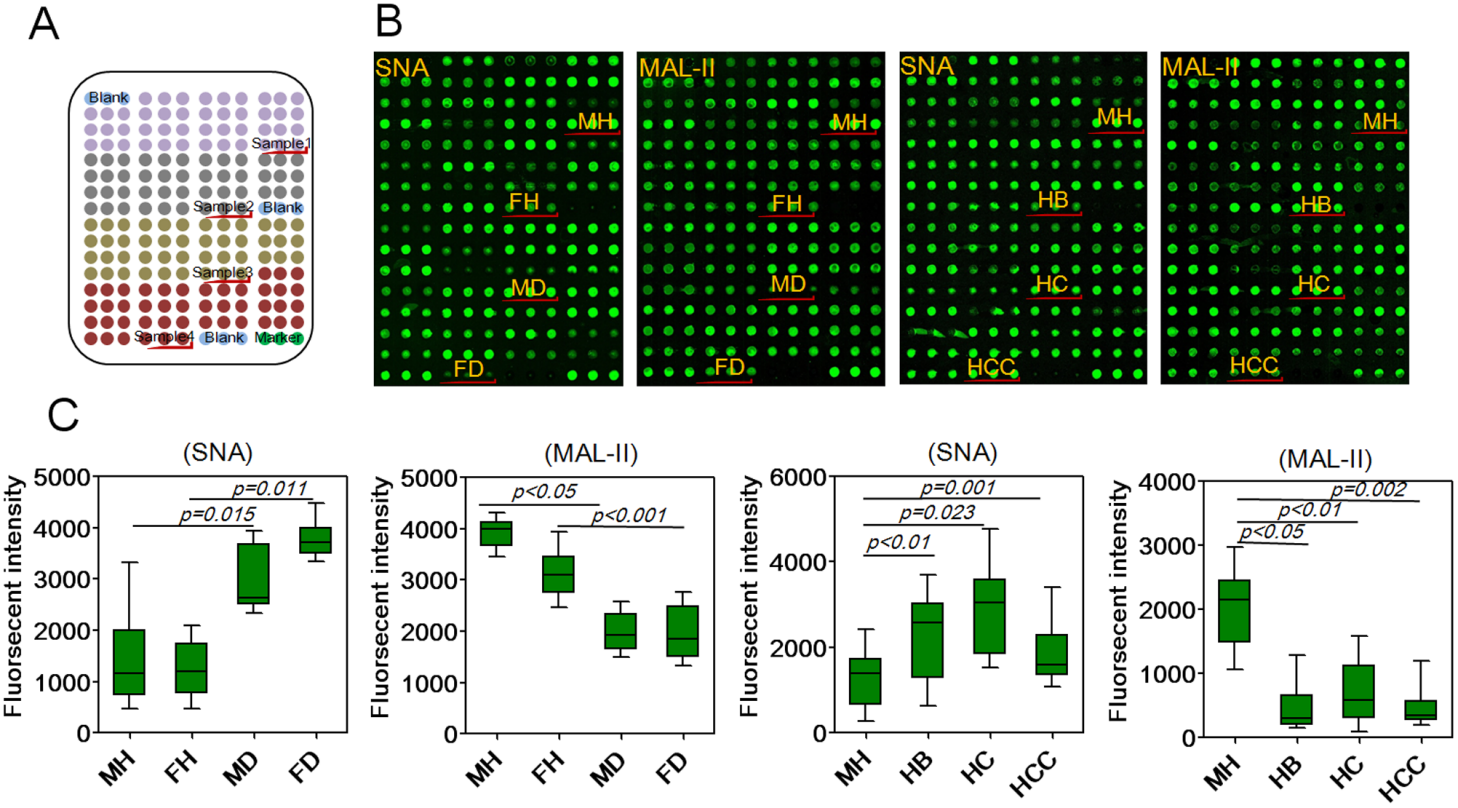
Validation of the expression levels of the terminal α2-3/6-linked sialic acids in individual salivary samples. (A) The layout of the salivary microarrays. A total of 210 individual salivary samples from male healthy group (MH, n = 30), female healthy group (FH, n = 30), male T2DM patients (MD, n = 30), female T2DM patients (FD, n = 30), as well as patients with HB (n = 30), HC (n = 30) and HCC (n = 30). Each sample was spotted in triplicate per block with two continuous blocks on one slide. (B) Scan images of Cy3-labeled SNA and MAL-II binding to the salivary microarrays. (C) Box plot analysis of the original data achieved from the salivary microarrays. Error bars represent 95% confidence intervals for means. Statistical significance of differences between groups was indicated by the *p*-value.

To further validate the expression levels of the terminal α2-3/6-linked sialic acids in individual saliva samples, the salivary microarrays was used to detect the terminal α2-3/6-linked sialic acids in individual samples from healthy groups and patients with T2DM, HB, HC and HCC (Figure 2A). The images and fluorescent intensities (FIs) were shown in Figure 2B and 2C. As a result, SNA staining showed increased the FIs (*p* < 0.05) in female elderly patients with T2DM and the male elderly patients with HC compared with healthy individuals, while MAL-II staining showed decreased the FIs (*p* < 0.05) in all elderly patients with T2DM and liver disease. Moreover, SNA staining showed that the expression levels of the terminal Siaα2-6Gal in male elderly patients with T2DM, HCC had no significant alternation. These results were basically coincident with the results from the lectin microarrays.

### Lectin blotting analysis

**Figure 3:**
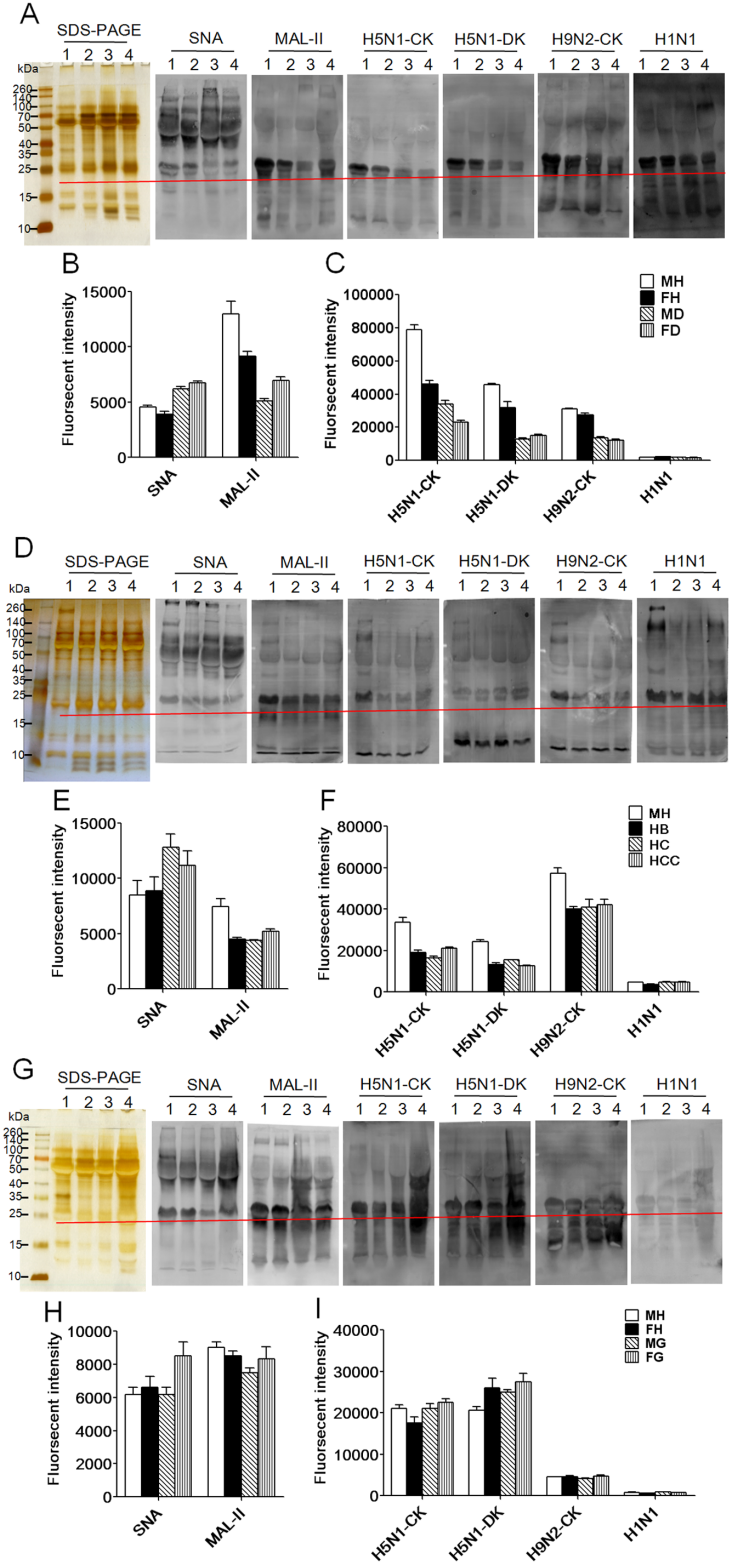
Validation of the terminal Siaα2-6Gal recognized by SNA and Siaα2-3Gal recognized by MAL-II in all salivary groups and a comparison of the binding pattern profiles of all salivary groups against three IAV strains A/Chicken/Guangxi/4/2009(H5N1), (A/Duck/Guangdong/17/2008(H5N1), A/Chicken/Fujian/S-1-521/2008(H9N2)) and the H1N1 influenza A vaccine. The summed fluorescence intensities (SFIs) of the bands marked by red lines at approximately 25 kDa for all salivary groups from lane 1 to lane 4 were read by ImageQuant Tools. (A) Lane 1: male healthy group (MH); lane 2: female healthy group (FH); lane 3: male T2DM patients (MD); lane 4: female T2DM patients (FD). (B) The summed fluorescence intensities for SNA and MAL-II strains binding to samples from the T2DM salivary group. (C) The SFIs of the bands marked by red lines at approximately 25 kDa for three IAV strains and the H1N1 vaccine strains binding to the salivary protein samples from the T2DM group. (D) Lane 1: MH; lane 2: hepatitis B (HB); lane 3: hepatic cirrhosis (HC); lane 4: hepatocellular carcinoma (HCC). (E) The SFIs of the bands marked by red lines at approximately 25 kDa for SNA and MAL-II strains binding to the salivary protein samples from the group with liver disease. (F) The SFIs of the bands marked by red lines at approximately 25 kDa for three IAV strains and the H1N1 vaccine strains binding to the salivary protein samples from the group with liver disease. (G) Lane 1: MH; lane 2: FH; lane 3: male GC patients (MG); lane 4: female GC patients (FG). (H) The summed fluorescence intensities for SNA and MAL-II strains binding to the salivary protein samples from the group with GC. (I) The SFIs of the bands marked by red lines at approximately 25 kDa for three IAV strains and the H1N1 vaccine strains binding to the salivary protein samples from the group with GC.

To confirm the different abundance of the terminal Siaα2-6Gal and Siaα2-3Gal between each clinical group and healthy group blotting analyses were performed with SNA and MAL-II (Figure 3A, 3D and 3G) The SDS-PAGE showed that the salivary proteins from all groups were similar in their molecular weight, and the different apparent bands in the healthy groups are distinguished from the clinical groups at approximately 10 kDa and between 100 kDa and 260 kDa. The results of the Siaα2-6Gal/GalNAc binder SNA and the Siaα2-3Galβ1-4Glc(NAc)/Glc binder MAL-II blotting analysis showed that the different apparent bands belong to a molecular weight range of 10 kDa to 260 kDa for all groups. However, the SNA apparent band between 25 kDa and 260 kDa for the healthy groups was distinguished from the clinical groups. The MAL-II apparent band between 10 kDa and 260 kDa for the healthy groups was distinguished from the clinical groups. The summed fluorescence intensities (SFIs) of the band marked by a red line at approximately 25 kDa for all salivary groups from lane 1 to lane 4 were shown in Figure 3B, 3E and 3H. The SFIs of SNA and MAL-II from each clinical group were compared with the healthy group. According to the results, the expression level of the terminal Siaα2-6Gal of all elderly groups with T2DM and liver disease were up-regulated, and the terminal Siaα2-3Gal of all elderly groups with T2DM and the male elderly groups with liver disease were down-regulated. However, the expression level of the terminal Siaα2-6Gal of the male elderly group with GC had no significant alteration, and the expression level of the terminal Siaα2-6Gal of the female elderly group with GC was up-regulated. The terminal Siaα2-3Gal of all elderly groups with GC was not significantly altered in the saliva. The blotting analyses of SNA and MAL-II to all salivary groups agreed with the results of the lectin microarrays.

### Comparison of binding of saliva against Influenza A Virus

The binding activity of saliva from each clinical group and healthy group was assessed against three strains (A/Duck/Guangdong/17/2008(H5N1), A/Chicken/Guangxi/4/2009 (H5N1), A/Chicken/Fujian/S-1-521/2008(H9N2)) of AIV and H1N1 influenza A vaccine^24^,^25^ by blotting analyses (Figure 3A, 3D and 3G). The resulting binding patterns were similar with that of MAL-II blotting. Three strains of AIV and H1N1 vaccine showed the strongest binding to a glycoprotein band at approximately 25 kDa for all salivary groups. The SFIs of the band at approximately 25 kDa for all salivary groups from lane 1 to lane 4 were shown in Figure 3C, 3F and 3I. According to these results, three AIV strains showed stronger band binding at approximately 25 kDa for the healthy groups than all of the elderly groups with T2DM and the male elderly groups with liver disease, and there was no significant difference between the healthy groups and all of the elderly groups with GC. The H1N1 influenza A vaccine presented almost the same binding at approximately 25 kDa for all salivary groups. This is because the H1N1 influenza A vaccine was developed by a seed virus prepared from reassortant vaccine virus A/California/7/2009 NYMC X-179A, including avian, swine and human flu and three types of influenza virus RNA gene fragments^26^. The results revealed that elderly individuals with chronic diseases, such as diabetes and liver disease, were more susceptible to AIV due to the decreased salivary expression of α2-3-linked sialic acid residues compared with the healthy groups.

### Binding of saliva to Influenza A Virus

**Figure 4:**
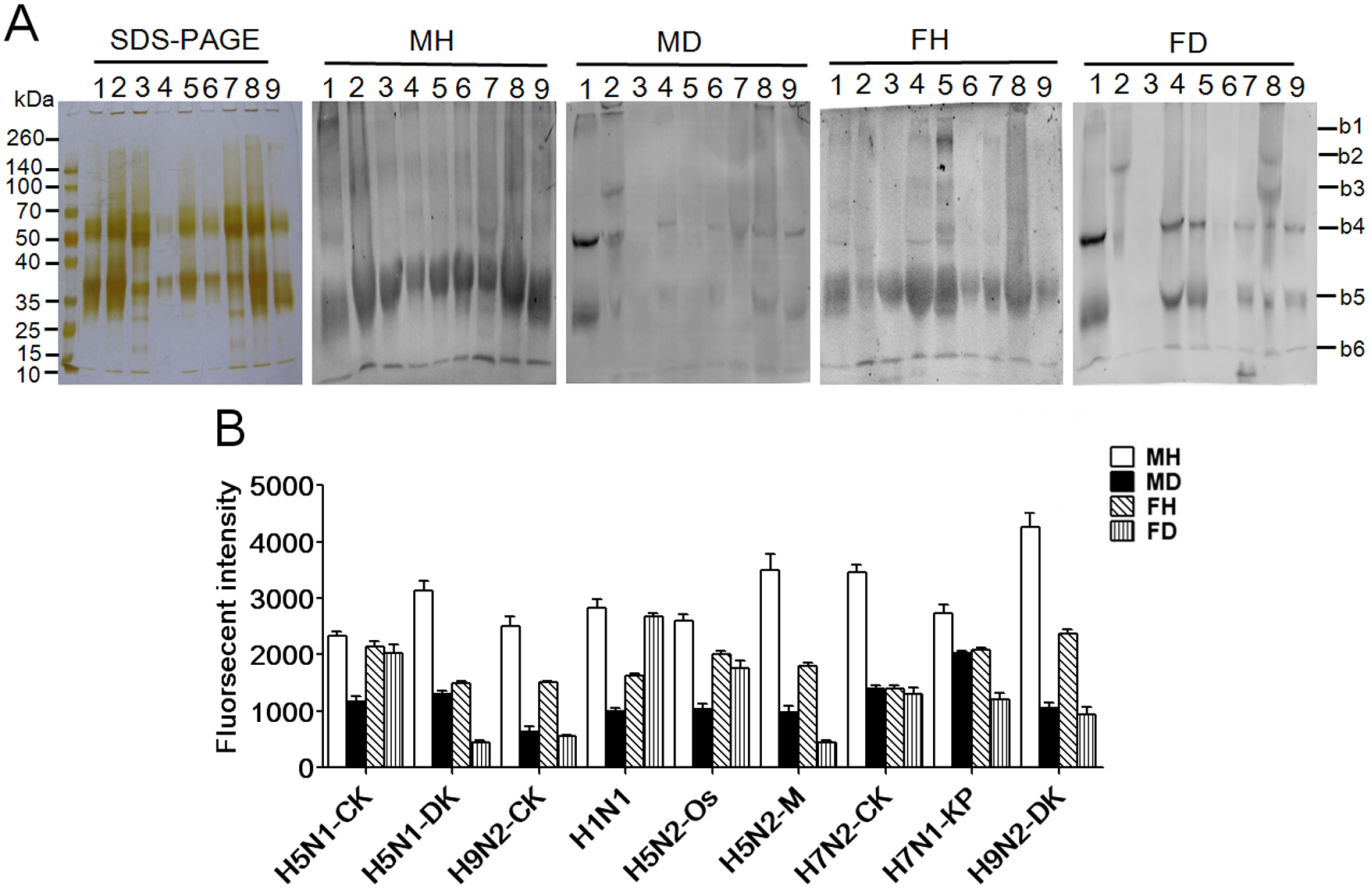
Comparison of the binding pattern profiles of eight IAV strains and the H1N1 influenza A vaccine against the binding patters of salivary groups with T2DM. MH: male healthy group, MD: male T2DM patients, FH: female healthy group, FD: female T2DM patients; The summed fluorescence intensities of the blotting analyses for IVA; lane 1 to lane 9 were read by ImageQuant Tools. (A) Lane 1: A/Chicken/Guangxi/4/2009(H5N1); Lane 2: A/Duck/Guangdong/17/2008(H5N1); Lane 3: A/Chicken/Fujian/S-1-521/2008(H9N2); Lane 4: H1N1 influenza A vaccine; Lane 5: A/Ostrich/Denmark/96-72420/1996(H5N2); Lane 6: A/Mallard/Jiangxi/16/2005(H5N2); Lane 7: A/Chicken/Hebei/1/2002(H7N2); Lane 8: A/Fowl/Rostock/45/1934(H7N1); Lane 9: A/Duck/Guangdong/S-7-134/2004 (H9N2). (B) The summed fluorescence intensities for the salivary samples from the T2DM group that bound to 8 IAV strains and the H1N1 vaccine.

To compare the binding pattern profiles of the eight strains of AIV and the H1N1 influenza A vaccine against the salivary groups with T2DM, blotting analyses were performed. Figure 4A showed the SDS-PAGE results for the eight strains of AIV and the H1N1 influenza A vaccine; the molecular weights were similar, and apparent bands between 70 kDa and 50 kDa as well as between 25 kDa and 40 kDa were presented. The blotting analysis results of the eight AIV strains showed stronger binding to a band between 25 kDa and 40 kDa for the healthy groups than for all of the elderly groups with T2DM. However, the results of the blotting analysis of the H1N1 influenza A vaccine showed stronger binding to a band between 25 kDa and 40 kDa for the male healthy group and weaker binding for female healthy group than the binding observed for the male and female clinical groups with T2DM, respectively. The SFIs of the band between 25 kDa and 40 kDa for the eight strains of AIV and the H1N1 influenza A vaccine were shown in Figure 4B (lane 1 to lane 9). This result also showed that elderly individuals with T2DM have fewer α2-3-linked sialic acid residues in their saliva compared with healthy individuals, possibly explaining one of the molecular events involved in the susceptibility of elderly individuals with T2DM to AIV.

## Discussion

Chronic disease conditions are associated with serious influenza-related complications, including elevated mortality. A previous study has shown that chronic obstructive pulmonary disease (COPD), asthma, arthritis, asthma, cancer, diabetes, liver disease, HIV/AIDS, neurological diseases, and heart and lung diseases can weaken a patient's immune system against the flu, placing patients at an increased risk of flu-related complications^21^,^23^,^27^,^28^. Here, we observed that the expression levels of the terminal α2-3-linked sialic acids in elderly individuals with T2DM and liver disease were down-regulated significantly and that the terminal α2-6-linked sialic acids were up-regulated slightly or had no significant alteration. However, both of these sialic acids were not significant altered in the saliva of patients with GC. Our studies provide evidence that elderly individuals with chronic diseases, such as diabetes and liver disease, might be more susceptible to avian influenza virus due to the decreased expression level of terminal α2-3-linked sialic acids and that these elderly individuals have stronger/normal resistance to human influenza viruses partly by presenting more terminal α2-6-linked sialic acids in their saliva. However, the patients with GC have normal resistance to human/avian influenza viruses similar to the healthy elderly groups. Our findings imply that the expression level alterations of terminal α2-3/6-linked sialic acids is a risk factor that could be a biomarker to distinguish those patients who are at a greater risk for infection with IAV, and may provide pivotal information to recommend strongly routine vaccination for them with influenza vaccines.

Our analyses have three limitations. First, we have investigated the expression level of the terminal α2-3- and α2-6-linked sialic acids in human saliva only from T2DM, liver disease and GC patients. Many chronic diseases (especially such as cardiovascular diseases and chronic respiratory diseases) that can make patients at an increased risk of flu-related complications need to be researched further^21^,^23^,^27^,^28^. Second, our investigation has not referred to the mechanisms that cause the decreased expression of terminal α2-3-linked sialic acids in saliva of elderly individuals with chronic diseases. Another limitation of our study is that anti-influenza activity is not assessed. A greater expression of either α2-3- and α2-6-linked sialic acids does not provide evidence of greater antiviral activity. Blotting studies showed binding of influenza virus to saliva proteins, but again a functional measure can strengthen the results.

In conclusion, we have reported the expression level alterations of terminal α2-3/6-linked sialic acids, which is a risk factor that could be a biomarker to distinguish those patients who are at a greater risk for infection with IAV. Our studies provide evidence that elderly individuals with chronic diseases, such as diabetes and liver disease, might be more susceptible to AIV. Currently, AIV outbreaks among poultry occur worldwide from time to time. In fact, hospitalizations and deaths after an influenza infection mainly occur in at-risk groups: the elderly population living with chronic diseases. Our findings will help understand this important epidemiological phenomenon, and distinguish exactly those patients who are at a greater risk for infection with IAV. Our framework could be inspire new ideas to facilitate improved prevention for the susceptible population of IVA by considering the individual conditions based on the salivary analysis firstly, and inform risk assessment and health policy for the elderly population living with chronic diseases at any time.

## Methods

### Ethics statements

The collection of human whole saliva was carried out in accordance with the approved guidelines, approved by the Human Ethics Committee of all participating units (Northwest University, Harbin Veterinary Research Institute, Fourth Military Medical University, Shaanxi Provincial People's Hospital, Academy of Military Medical Science and University of South China), and informed consent was received from each participant. The clinical and demographic characteristics of T2DM, liver disease (HB, HC, HCC) and GC patients as well as the healthy groups were summarized in Supplementary Table S1, S2 and S3. The non-smoking and chronically healthy groups were screened for good general and oral health with normal salivary function.

### Whole saliva preparation and protein labeling

Unstimulated saliva was collected between 9 a.m. and 10 a.m., at least 2 h after the last intake of food, from patients with T2DM, HB, HC, HCC and GC and from the healthy groups according to the protocol^20^,^29^,^30^. The mouth was rinsed with physiological saline immediately before collection. Whole saliva (approximately 1 mL) was collected and placed on ice. Protease Cocktail Inhibitor (1 μL/mL of whole saliva) was added to the saliva immediately after collection to minimize protein degradation.

Whole saliva was then centrifuged at 12 000 rpm at 4°C for 30 min to remove the insoluble materials. The supernatant was collected and filtered with a 0.20-μm pore size to remove bacteria and microbials and then immediately used or stored at −80°C. To normalize the differences between subjects and to account for individual variation, 100 μL from each saliva sample was pooled in each group, the other maintained for further validation. The pooled saliva protein concentration of the T2DM, HB, HC, HCC and GC groups as well as the healthy groups determined by the Bradford assay (Supplementary Figure S1) were listed in Supplementary Table S1, S2 and S3. The pooled salivary proteins were then labeled with Cy3 fluorescent dye (GE Healthcare, Buckinghamshire, UK) and purified using Sephadex G-25 columns according to the manufacturer's instructions.

### Lectin microarrays and data analysis

A lectin microarray was produced by using 37 lectins (purchased from Vector Laboratories, Sigma-Aldrich and Calbiochem) with different binding preferences covering *N*- and *O*-linked glycans. The lectins were dissolved to a concentration of 1 mg/mL in the manufacturer's recommended buffer containing 1 mmol/L appropriate monosaccharide and spotted onto the homemade epoxysilane-coated slides, according to the protoco1, with Stealth micro spotting pins (SMP-10B) (TeleChem, USA) by a Capital smart microarrayer (CapitalBio, Beijing)^31^,^32^. Each lectin was spotted in triplicate per block, with triplicate blocks on one slide (Supplementary Figure S2). The slides were incubated in a humidity-controlled incubator at 50% humidity overnight and then put into a vacuum dryer for 3 h at 37°C to allow lectin immobilization. After incubation, the slides were blocked with blocking buffer containing 2% (w/v) BSA in 1 × PBS (0.01 mol/L phosphate buffer containing 0.15 mol/L NaCl, pH7.4) for 1 h, then rinsed twice with 1 × PBST (0.2% Tween 20 in 0.01 mol/L phosphate buffer containing 0.15 mol/L NaCl, pH 7.4), and followed by a final rinse in 1 × PBS. Afterwards, 4 μg of Cy3-labeled salivary protein was diluted in 0.5 mL of incubation buffer containing 2% (w/v) BSA, 500 mmol/L glycine and 0.1% Tween-20 in 1 × PBS and was applied to the blocked lectin microarrays and an incubation was performed in the chamber at 37°C for 3 h in a rotisserie oven set at 4 rpm. Each slide was washed twice with 1 × PBST for 5 min, washed once with 1 × PBS for 5 min, and then dried by centrifugation at 600 rpm for 5 min. The microarrays were scanned with a 70% photomultiplier tube and 100% laser power settings using a Genepix 4000B confocal scanner (Axon Instruments, USA). The acquired images were analyzed at 532 nm for Cy3 detection by Genepix 3.0 software (Axon Instruments, Inc. USA). The average background was subtracted, and the values less than average background ±2 standard deviations (SD) were removed from each data point. The median of the effective data points of each lectin was globally normalized to the sum of medians of all effective data points for each lectin in one block. Each sample was observed consistently by three repeated slides, and the normalized medians of each lectin from 9 repeated blocks were averaged and its SD was calculated. The normalized data of each clinical group were compared with the healthy groups based upon the fold change according to the following criteria: fold change >1.5 or <0.67 in the pairs indicated up-regulation or down-regulation, respectively, of a certain glycan. Differences between the arbitrary two data sets or multiple data sets were tested by Student's *t*-test or one-way ANOVA to each lectin signal using SPSS statistics 19 software.

### Salivary microarrays

A saliva microarray was produced according to our previous protocols with some modifications^20^. Totally, 210 saliva individual samples from healthy groups and patients with T2DM and liver disease were dissolved in spotting buffer containing 0.5 mg/mL BSA in 1 × PBS, pH 7.4, to a concentration of 1 mg/mL and spotted on the homemade epoxysilane-coated slides with Stealth micro spotting pins (SMP-10B) by a Capital smart microarrayer. Each saliva sample was spotted in triplicate and the layout of the saliva microarrays was shown in Figure 2A. After immobilization, the slides were blocked with the buffer (0.15 mol/L NaCl and 0.5 mg/mL BSA in 10 mmol/L HEPES, pH 7.5) for 1 h and rinsed twice with 1 × PBS. Cy3-labeled lectin diluted in 0.5 mL of the same buffer was incubated with the blocked slide for 3 h at room temperature in the dark. The slide was washed and dried by centrifugation at 600 rpm for 5 min. Then the slide was scanned using a Genepix 4000B confocal scanner and the acquired images were analyzed at 532 nm for Cy3 detection. The values less than average background ±2SD were removed from each data point and the median of the effective data points of each sample was counted. Differences between the arbitrary two groups or multiple groups of medians were tested by Student's *t*-test or one-way ANOVA to each saliva samples using SPSS statistics 19.

### SDS-PAGE and lectin blotting

The pooled salivary proteins of each group were analyzed by SDS-PAGE, and subsequently, lectin blotting was performed according to the protocol^20^. SDS-PAGE samples were boiled for 4 min at 100°C, mixed with 5× loading buffer and run on a 10% polyacrylamide resolving gel and a 3% stacking gel. Molecular mass standards (Thermo Scientific, Waltham, USA) were run with all gels. Some gels were then stained directly with alkaline silver. For lectin blotting, the proteins in the gels were then transferred to a PVDF membrane (Millipore Corp., USA) with a wet transfer unit (Hoefer Scientific, USA) for 1.5 h at 100 V. After transfer, the membranes were washed twice with TTBS (150 mmol/LNaCl, 10 mmol/LTris-HCl, 0.05% Tween-20, pH 7.5) and then blocked for 1 h with Carbo-Free Blocking Solution (Vector, Burlingame, CA) at room temperature. On the basis of silver staining of the gels after transfer, it was evident that lower-molecular-mass proteins (less than 50 kDa) transferred more thoroughly to the blots. Hence, if any bias was present in the final results, it favored the detection of proteins smaller than 50 kDa during lectin blotting. The membranes were then washed again and incubated with Cy5-labeled (GE Healthcare, Buckinghamshire, UK) MAL-II and SNA lectins (2 μg/mL in Carbo-Free Blocking Solution) with gentle shaking overnight at 4°C in the dark. The membranes were then washed twice each for 10 min with TTBS and scanned on the red fluorescence channel (635 nm excitation/650 nm LP emission) with a voltage of 800 PMT using a phosphorimager (Storm 840, Molecular Dynamics Inc. USA).

### Influenza A Virus preparation

Two H5N1 subtype strains (A/Duck/Guangdong/17/2008, A/Chicken/Guangxi/4/2009), two H5N2 subtype strains (A/Mallard/Jiangxi/16/2005, A/Ostrich/Denmark/96-72420/1996), one H7N1 subtype strain (A/Fowl/Rostock/45/1934), one H7N2 subtype strain (A/Chicken/Hebei/1/2002) and two H9N2 subtype strains (A/Duck/Guangdong/S-7-134/2004 and A/Chicken/Fujian/S-1-521/2008) were cultured in the chorioallantoic fluid of 10-day-old embryonated hen eggs and then purified on a discontinuous sucrose-density gradient as described previously^20^,^33^,^34^.

### Assessment of binding of Influenza A Virus to saliva

Viral proteins were extracted with a mixture of ether and ethanol, the aqueous and organic phases were separated, and the residual ether was removed. The extracted proteins were labeled with Cy5 fluorescent dye and purified with Sephadex G-25 columns according to the manufacturer's instructions. The pooled saliva samples from each group were subjected to 10% SDS-PAGE and transferred to PVDF membranes. The membranes were blocked with Carbo-Free Blocking Solution and then incubated with Cy5-labeled viral proteins (2 μg/mL in Carbo-Free Blocking Solution) of H5N1, H9N2 and H1N1 influenza A vaccine (Split Virion, inactivated) (Sinovac Biotech Ltd., Beijing)^20^. Finally, the membranes were scanned using the red fluorescence channel (635 nm excitation/650 nm LP emission) with a voltage of 800 PMT using a phosphorimager and further analyzed by ImageQuant Tools.

### Assessment of binding of saliva to Influenza A Virus

The pooled saliva samples from the patients with T2DM and from the healthy groups were labeled with Cy5 fluorescent dye and purified with Sephadex G-25 columns according to the manufacturer's instructions. The extracted viral proteins were subjected to 10% SDS-PAGE and transferred to PVDF membranes. The membranes were blocked with Carbo-Free Blocking Solution and then incubated with Cy5-labeled salivary proteins (2 μg/mL in Carbo-Free Blocking Solution). Finally, the membranes were scanned using the red fluorescence channel (635 nm excitation/650 nm LP emission) with a voltage of 800 PMT using a phosphorimager and further analyzed by ImageQuant Tools.

## Author Contributions

Y.Z., Y.Q., H.Y. and Z.L. designed the experiments. Y.Z., Y.Q., H.Y., J.Y., H.W., L.C. and P.Z. performed the experiments. X.W. provided eight strains of Influenza A virus samples. J.S. and Y.W. provided H1N1 influenza A vaccine. Z.J., Y.G., H.Z., H.X. and X.L. collect the saliva of type 2 diabetes mellitus, liver disease, gastric cancer patients and the healthy groups. Y.Z. wrote the first draft and all authors contributed to review and revision and have seen and approved the final version.

## Supplementary Material

Supplementary Information Supplementary information
