# Supplementary material for: Avian Influenza Virus Infection Risk in Humans with Chronic Diseases
Source: Sci Rep. 2015 Mar 10;5:8971. doi: 10.1038/srep08971 (PMC4354171; doi:10.1038/srep08971)
Supplement: Supplementary Information [file srep08971-s1.pdf]

Supplementary information for

Avian Influenza Virus Infection Risk in Humans with Chronic Diseases

Yaogang Zhong[1]†, Yannan Qin[1]†, Hanjie Yu[1]†, Jingmin Yu[1], Haoxiang Wu[1], Lin Chen[1], Peixin Zhang[1], Xiurong Wang[2], Zhansheng Jia[3], Yonghong Guo[3], Hua Zhang[4], Junjie Shan[5], Yuxia Wang[5], Hailong Xie[6], Xiaojie Li[6], Zheng Li[1]*

[1]Laboratory for Functional Glycomics, College of Life Sciences, Northwest University, Xi'an, PR China.

[2]National Key Laboratory of Veterinary Biotechnology, Harbin Veterinary Research Institute, Chinese Academy of Agricultural Science, Harbin, PR China.

[3]Center of infectious diseases, Tangdu Hospital, Fourth Military Medical University, Xi’an, PR China.

[4]Department of Oncology, Shaanxi Provincial People’s Hospital, Xi’an, PR China.

[5]Institute of Pharmacology and Toxicology, Academy of Military Medical Science, Beijing, PR China.

[6]Cancer Research Institute, University of South China, Hengyang, PR China.

†These authors contributed equally to this work.

*Corresponding authors. E-mail: zhengli@nwu.edu.cn

This PDF file includes:

Supplementary Figure S1 to S2

Supplementary Table S1 to S3

Supplementary Figures and Tables :

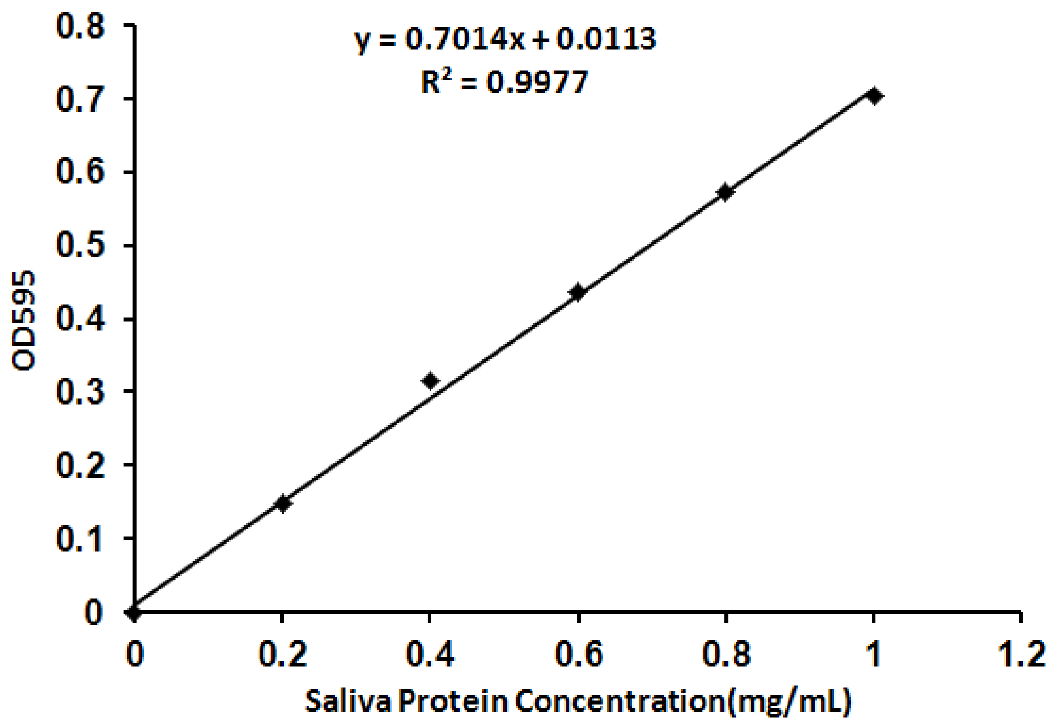

Supplementary Figure S1. (A) Whole Saliva Protein Concentration. The whole saliva protein concentrations of T2DM, HB, HC, HCC and GC patients as well as healthy groups were measured using the Bradford assay.

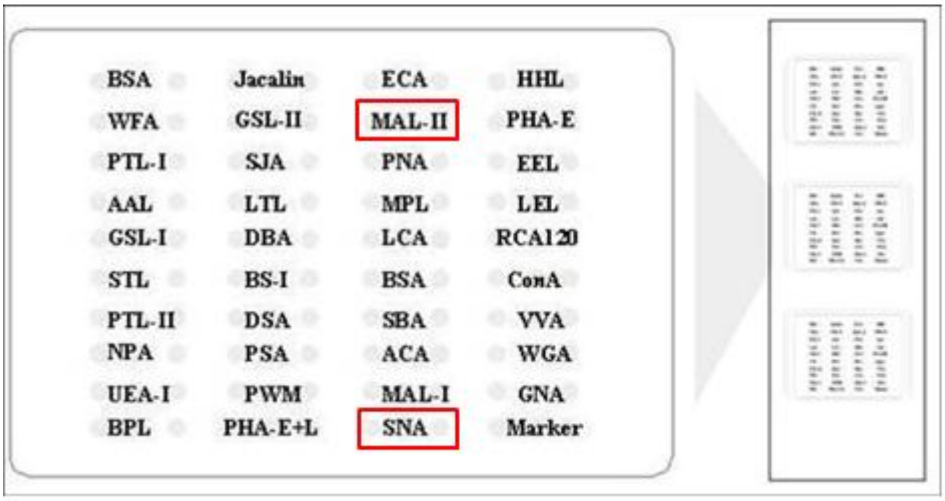

Supplementary Figure S2. The layout of Lectin microarray.

Supplementary Table S1. The clinical and demographic characteristics of T2DM patients and healthy groups.

Parameter			Healthy groups	T2DM	Bradford assay			
					OD_{595}		PC (mg/mL)	
Sample number(n)			60	64	H	D	H	D
Gender	male		50	30	0.546	0.596	0.752	0.834
	female		50	34	0.523	0.645	0.730	0.903
Age, years, median, SD			57±10.3	57±11.3				
HbA1c(%)	4-6		60(100%)	—				
	>9		—	28(43.8%)				
	>10		—	36(56.2%)				
OGTT(mmol/L)	0.5-1h	7.8-9.0	Negative	Positive				
	2h	<7.8						
	>3h	3.9-6.1						
FBG (mmol/L)			3.9-6.1	6.8-11				
Lectin	specificity		Male, NFI±SD[a]		Female, NFI±SD[a]		Fold change	
			MH	MD	FH	FD	MD/MH	FD/FH
SNA	Siaα2-6Gal/GalNAc		0.0140 ±0.0030	0.0200 ±0.0041	0.0131 ±0.0010	0.0232 ±0.0070	1.43*	1.77**
MAL-II	Siaα2-3Galβ1-4Glc(NAc)/Glc, Siaα2-3Gal, Siaα2-3, Siaα2-3GalNAc		0.0141 ±0.0040	0.0041 ±0.0090	0.0120 ±0.0010	0.0071 ±0.0042	0.291***	0.592*

H: healthy groups, D: T2DM patients, MH: male healthy groups, FH: female healthy groups, MD: male T2DM patients, FD: female T2DM patients, a:Normalized fluorescent intensities (NFI) obtained for 9 repeated blocks in three repeated slides were averaged and its SD was counted. PC: Protein Concentration, $^{*}P<0.05$, $^{**}P<0.01$, $^{***}P<0.001$

Supplementary Table S2. The clinical and demographic characteristics of HB, HC, and HCC patients and healthy groups.

Parameter		Healthy groups	HB	HC	HCC	Fold change		
Sample number (n) /male		33	36	37	32	HB/H	HC/H	HCC/H
Age, years, median, SD		65±7.2	62±9.5	61±11.7	65±7.6			
Bradford assay	OD_{595}	0.549	0.676	0.658	0.698			
	PC(mg/mL)	0.767	0.945	0.922	0.979			
Log (HBV DNA) (copies/mL)		—	6.84 ±2.02	5.17 ±3.77	4.21 ±1.18			
ALT(IU/mL), median, (range)		0-40	110 (10.3-58.8)	30.3 (27.4-157)	23.2 (33.0-127)			
AST(IU/mL), median, (range)		0-40	73.5 (14.1-64.4)	55.6 (18.3-126)	52.4 (24.1-254)			
AFP(μg/L), median, (range)		0-25	36.1 (7.50-29.8)	89.5 (27.6-104)	584 (17.0-271)			
Lectin	specificity	NFI±SD[a]						
SNA	Siaα2-6Gal/GalNAc	0.0139 ±0.0090	0.0318 ±0.0011	0.0196 ±0.0003	0.0185 ±0.0020	2.29**	1.41**	1.33*
MAL-II	Siaα2-3Galβ1-4Glc(NAc)/Glc, Siaα2-3Gal, Siaα2-3, Siaα2-3GalNAc	0.0089 ±0.0005	0.0038 ±0.0008	0.0039 ±0.0007	0.0043 ±0.0040	0.427*	0.438***	0.483*

HB: Hepatitis B patients, HC: Hepatic cirrhosis patients, HCC: Hepatocellular carcinoma patients, a:Normalized fluorescent intensities (NFI) obtained for 9 repeated blocks in three repeated slides were averaged and its SD was counted, PC: Protein Concentration, $^{*}P<0.05$, $^{**}P<0.01$, $^{***}P<0.001$

Supplementary Table S3. The clinical and demographic characteristics of GC patients and healthy groups.

Parameter		Healthy groups	GC	Bradford assay			
				OD_{595}		PC (mg/mL)	
Sample number(n)		64	70	H	G	H	G
Gender	male	30	30	0.493	0.620	0.687	0.868
	female	34	40	0.528	0.646	0.737	0.905
Age, years, median, SD		57±11.6	57±11.3				
Tumor size (cm)	<2	—	18(25.0%)				
	2-5		29(41.7%)				
	>5		23(33.3%)				
TMN classification	Ⅰ, Ⅱ	—	27(38.9%)				
	Ⅲ, Ⅳ		43(61.1%)				
Microvascular invasion	NO	—	49(69.4%)				
	Yes		21(30.6%)				
Histology	Well	—	19(27.8%)				
	Moderate	—	45(63.9%)				
	Poor	—	6(8.3%)				
Lectin	specificity	NFI±SD[a]				Fold change	
		MH	MG	FH	FG	MG/MH	FG/FH
SNA	Siaα2-6Gal/GalNAc	0.0415 ±0.0008	0.0325 ±0.0020	0.0370 ±0.0091	0.0400 ±0.0020	0.783^{*}	1.08^{***}
MAL-II	Siaα2-3Galβ1-4Glc(NAc)/Glc, Siaα2-3Gal, Siaα2-3, Siaα2-3GalNAc	0.0345 ±0.0031	0.0256 ±0.0042	0.0320 ±0.0051	0.0288 ±0.0031	0.742^{**}	0.900^{*}

MH: male healthy groups, FH: female healthy groups, MG: male GC patients, FG: female GC patients, a: Normalized fluorescent intensities (NFI) obtained for 9 repeated blocks in three repeated slides were averaged and its SD was counted, PC: Protein Concentration, $^{*}P<0.05$, $^{**}P<0.01$, $^{***}P<0.001$
